# Adenocarcinoma with mixed subtypes is a rare but aggressive histologic subtype in colorectal cancer

**DOI:** 10.1186/s12885-019-6245-5

**Published:** 2019-11-08

**Authors:** Hui Sheng, Xiaoli Wei, Minjie Mao, Jincan He, Tianqi Luo, Shilin Lu, Liye Zhou, Zhixin Huang, Anli Yang

**Affiliations:** 1 Department of Experimental Research, Sun Yat-sen University Cancer Center, State Key Laboratory of Oncology in South China, Collaborative Innovation Center for Cancer Medicine, Guangzhou, 510060 China; 2 Department of Medical Oncology, Sun Yat-sen University Cancer Center, State Key Laboratory of Oncology in South China, Collaborative Innovation Center for Cancer Medicine, Guangzhou, 510060 China; 3 Department of Clinical Laboratory, Sun Yat-sen University Cancer Center, State Key Laboratory of Oncology in South China, Collaborative Innovation Center for Cancer Medicine, Guangzhou, 510060 China; 4 Zhongshan School of Medicine, Sun Yat-sen University, Guangzhou, 510008 China; 5 Department of Gastric Surgery, Sun Yat-sen university Cancer center, State key Laboratory of Oncology in South China, Collaborative Innovation Center for Cancer Medicine, Guangzhou, 510060 China; 6 Department of Medical Oncology, Dana-Farber Cancer Institute, Boston, MA USA; 7 Department of Emergency, Foshan Traditional Chinese Medicine Chancheng High-tech Zone Hospital, 10 Chunyang Road, Foshan, 52800 Guangdong Province China; 8 Department of Breast Oncology, Sun Yat-sen University Cancer Center, State Key Laboratory of Oncology in South China, Collaborative Innovation Center for Cancer Medicine, 651 Dong Feng Road East, Guangzhou, 510060 Guangdong Province China

**Keywords:** Colorectal cancer, Histologic subtypes, Prognosis

## Abstract

**Background:**

Although numerous studies have investigated the clinicopathologic and prognostic relevance of mucinous adenocarcinoma (MAC) and signet-ring cell carcinoma (SRCC) compared with classic adenocarcinoma (CA), little is known about the prognosis of adenocarcinoma with mixed subtypes (AM) and the differences among these four subtypes.

**Methods:**

The statistics of colorectal cancer registered in the Surveillance, Epidemiology and End Results (SEER) database were retrieved and analyzed. We also compared the clinicopathologic and prognostic relevance between CA, SRCC, MAC, and AM.

**Results:**

The frequencies of these four subtypes were 69.9% (CA, *n* = 15,812), 25.1% (MAC, *n* = 5689), 3.6% (SRCC, *n* = 814) and 1.4% (AM, *n* = 321), respectively. All of MAC, SRCC, and AM were significantly related with aggressive features. Only SRCC and AM were identified as independent poor prognostic markers for overall survival by multivariate analysis. The aggressiveness of AM was between MAC and SRCC according to the clinicopathologic associations. The prognosis of AM was significantly worse than MAC but comparable with SRCC.

**Conclusions:**

We confirmed the clinicopathologic relevance with aggressive features of MAC and SRCC, as well as poor prognostic relevance of SRCC by analyzing a large study population data set. Furthermore, we identified AM as a rare but aggressive histologic subtype in colorectal cancer, to which particular attention should be given in clinical practice.

## Background

Colorectal cancer (CRC) is the third most common malignancy in the US and the fifth in China [1, 2]. Early-stage CRC is curable by radical surgery. However, cancer recurrence and distant metastasis occur frequently after curative treatment, especially for more advanced stage CRC patients, which leads to poor outcomes [3–8]. Thus, the identification of prognostic markers is of great importance in patient management and decision making.

American Joint Committee on Cancer / Tumor-node-metastasis (AJCC/TNM) staging system is well accepted as the most efficient prognostic factor in CRC [9] However, heterogeneity of prognosis exists even among patients at the same TNM stage. Thus, it underlines the importance of incorporating multiple prognostic markers, such as tumor differentiation degree [10–13], some genetic markers [14, 15], and several postoperative pathologic features [16–18].

The histologic subtypes of CRC have also been demonstrated with prognostic relevance. Most CRCs are adenocarcinomas, including three well-studied major subtypes: classical adenocarcinoma (CA), mucinous adenocarcinoma (MAC), and signet-ring cell carcinoma (SRCC) [19]. MAC was primarily identified as a negative prognostic factor [20]. However, subsequent studies have proved that the prognostic difference between SRCC and MAC is not independently significant [19, 21–23]. Furthermore, in stage II CRC, MAC is associated with high microsatellite instability (MSI-H) [24, 25], a marker of superior prognosis and no benefit from adjuvant 5-Fu chemotherapy. Thus in the 3rd version of 2012 National Comprehensive Cancer Network (NCCN) guidelines for colorectal cancer, a poor differentiation with MSI-H was removed from the list of high-risk factors for stage II CRC. SRCC has been widely recognized as a marker of aggressive tumors with inferior prognosis [26, 27]. While there may still be other histologic subtypes with distinct clinicopathologic and prognosis relevance apart from CA that need special concern in clinical management.

We conducted this study with Surveillance, Epidemiology, and End Results Program (SEER) database for CRC registered during 2010–2012 to investigate the frequency distribution of histologic subtypes in CRC, and explore other possible histologic subtypes with distinct clinical significance compared with CA. Additionally, we sought to describe the impact of histological subtypes on prognosis.

## Methods

### The SEER database and cases selection

As the largest publicly available cancer dataset worldwide, the SEER database collects cancer information including morbidity, mortality, and disease status of patients with malignancies across the US. Unified and standardized tumor information in the database is updated regularly. Here we focused on colorectal adenocarcinomas, coded by the 3rd edition of the International Classification of Diseases for Oncology (ICD-O-3) as C18.0, C18.2 - C18.7, C19.9 and C20.9 for topography and 8140–8147, 8210–8211, 8220–8221, 8255, 8260–8263, 8480–8481, 8490 and 8574 for histology. In addition, the present study only covered patients with records of histologic codes (ICD-O-3), the 7^th^AJCC/TNM classification and follow-up information. Patients with other tumors as primary tumor were excluded.

### Histologic subtypes

The ICD-O-3 (updated in 2000) was used in tumor or cancer registries for coding the topography and histology. We summarized the histologic codes and the relevant corresponding descriptions in Additional file 1: Table S1. A further categorization was conducted to categorize patients into four histologic subtypes, including CA (Code 8141–8147, 8210–8211, 8220–8221, 8260–8263), MAC (Code 8480–8481), SRCC (Code 8490), and AM (Code 8255). Adenocarcinoma with neuroendocrine differentiation (Code 8574) was not included in the final analysis because of the difficulty in categorization and limited sample size.

### Statistical analysis

All the analyses were conducted with SPSS for Windows V.13.0. (SPSS Inc., Chicago, IL, USA). The frequency distribution of histologic subtypes was calculated with descriptive method. The comparisons of clinicopathologic characteristics between CA and the other histologic subtypes including MAC, SRCC, and AM were performed with chi-square test or Kruskal-Wallis H test. CRC-specific overall survival (OS) was the interval from the date of CRC diagnosis to the date of last follow-up or cause-specific death. Patients alive at the last follow-up or died of other causes were classified as censored cases. Univariate and multivariate analyses were performed for prognostic differences between histologic subtypes. Survival curves were plotted and compared using the Kaplan-Meier method and the log-rank test. A two-tailed *P* value < 0.05 was considered statistically significant. Variables with a *P* value < 0.05 in univariate analyses were included in multivariate analyses. We adopted “forward: conditional” method for multivariate analyses. With this method, only variables with a significant *P* value would be included for the estimation of hazard ratio (*HR*) 95% confidence interval (95% *CI*) in the Cox proportional hazards model.

## Results

### The frequency distribution of histologic subtypes in CRC

71,810 CRC patients were included in this study from SEER registers during 2010–2012. According to the ICD-O-3 codes and description, 49,131 (68.4%) cases were classified as adenocarcinoma NOS, not otherwise specified. The rest 22,679 (31.6%) patients were analyzed for the frequency distribution of histologic subtypes in CRC (Additional file 1: Table S1). Except for adenocarcinoma with neuroendocrine differentiation (Code 8574), which is a distinct subtype but accounts for a very small population, all the others (*n* = 22,636, 99.8%) were included and categorized into four histologic subtypes: CA, MAC, SRCC, and AM. The most common subtype was CA, with 15,812 cases accounting for 69.9%. The numbers and frequencies of MAC, SRCC, and AM were 5689 (25.1%), 814 (3.6%), and 321 (1.4%), respectively (Table 1). Both SRCC and AM are relatively rare with their frequencies lower than 5%.

**Table 1:** The frequency distribution of classical adenocarcinoma, mucinous adenocarcinoma, signet-ring cell carcinoma and adenocarcinoma with mixed subtypes in colorectal cancer

Histologic subtype	Number (%)
Classical adenocarcinoma	15,812 (69.9)
Mucinous adenocarcinoma	5689 (25.1)
Signet-ring cell carcinoma	814 (3.6)
Adenocarcinoma with mixed subtypes	321 (1.4)

### Comparisons of clinicopathologic differences between histologic subtypes

MAC was more common in female (*P* <  0.001) and older patients (*P* <  0.001) compared with CA. There was no significant difference in the distribution of gender and sex between CA and SRCC or AM. Compared with CA, all the other three subtypes were found less common in rectal cancer (all *P* <  0.001). In addition, MAC, SRCC, and AM, were significantly associated with some features of aggressiveness, including poor tumor differentiation, large size of primary tumors, high level of carcinoembryonic antigen (CEA), advanced T stage and N stage, distant metastasis, high positive rates of circumferential resection margin (CRM) involvement, and perineural invasion, as well as frequent presence of tumor deposits (all *P* <  0.001, Table 2, *P*1 using “CA” as the reference).

**Table 2:** Comparisons of the clinicopathologic features between classical adenocarcinoma and other histologic types, including mucinous adenocarcinoma, signet-ring cell carcinoma and adenocarcinoma with mixed subtypes

Characteristics (N)	Classical adenocarcinoma *N* (%)	Mucinous adenocarcinoma *N* (%)	*P*_1_^a^	*P*_2_^a^	Signet-ring cell carcinoma *N* (%)	*P*_1_^a^	*P*_2_^a^	Adenocarcinoma with mixed subtypes N (%)	*P*_1_^a^
Gender			< 0.001	0.05		0.11	0.33		0.89
Male	8511 (53.8)	2759 (48.5)			415 (51.0)			175 (54.2)	
Female	7301 (46.2)	2930 (51.5)			399 (49.0)			147 (45.8)	
Age (yrs, median: 66)			< 0.001	0.59		0.53	0.06		0.07
≤ 66	8894 (56.2)	2818 (49.5)			467 (57.4)			164 (54.5)	
> 66	6918 (43.8)	2871 (50.5)			347 (42.6)			157 (45.5)	
Tumor location			< 0.001	0.29		< 0.001	0.76		< 0.001
Colon	12,198 (77.1)	5055 (88.9)			713 (87.6)			279 (86.9)	
Rectum	3614 (22.9)	634 (11.1)			101 (12.4)			42 (13.1)	
Grade			< 0.001	< 0.001		< 0.001	< 0.001		< 0.001
Well differentiated	2308 (17.6)	705 (13.4)			8 (1.1)			4 (1.4)	
Moderately differentiated	9491 (72.6)	3344 (63.7)			58 (8.0)			53 (18.0)	
Poorly differentiated or undifferentiated	1278 (9.8)	1199 (22.8)			656 (90.9			237 (80.6)	
Primary tumor size (cm)			< 0.001	0.76		< 0.001	0.48		< 0.001
≤ 4	7754 (71.7)	1628 (31.3)			223 (32.8)			89 (30.5)	
> 4	3148 (28.9)	3568 (68.7)			457 (67.2)			203 (69.5)	
CEA			< 0.001	0.06		< 0.001	0.52		< 0.001
Normal	4804 (69.9)	1558 (46.4)			217 (42.4)			76 (39.2)	
Borderline	25 (0.4)	29 (0.9)			3 (0.6)			3 (1.5)	
Elevated	2043 (29.7)	1770 (52.7)			292 (57.0)			115 (59.3)	
T category			< 0.001	< 0.001		< 0.001	0.29		< 0.001
Tis	1988 (12.6)	21 (0.4)			4 (0.5)			0 (0.0)	
T1	7516 (47.5)	309 (5.4)			45 (5.5)			11 (3.4)	
T2	2168 (13.7)	547 (9.6)			34 (4.2)			21 (6.5)	
T3	3316 (21.0)	3120 (55.0)			341 (42.0)			150 (46.7)	
T4	822 (5.2)	1680 (29.6)			388 (47.8)			139 (43.3)	
N category			< 0.001	< 0.001		< 0.001	0.27		< 0.001
N0	12,776 (80.8)	3130 (55.0)			251 (30.8)			110 (34.3)	
N1	2244 (14.2)	1405 (24.7)			176 (21.6)			68 (21.2)	
N2	792 (5.0)	1154 (20.3)			387 (47.5)			143 (44.5)	
M category			< 0.001	0.002		< 0.001	0.01		< 0.001
M0	14,931 (94.4)	4505 (79.2)			519 (63.8)			231 (72.0)	
M1	881 (5.6)	1184 (20.8)			295 (36.2)			90 (28.0)	
CRM			< 0.001	< 0.001		< 0.001	0.60		< 0.001
Negative	5685 (85.7)	2163 (73.7)			220 (62.1)			101 (59.8)	
Positive	950 (14.3)	770 (26.3)			134 (37.9)			68 (40.2)	
Perineural invasion			< 0.001	< 0.001		< 0.001	0.003		< 0.001
Negative	12,234 (95.6)	3957 (89.7)			383 (70.8)			201 (80.7)	
Positive	569 (4.4)	454 (10.3)			158 (29.2)			48 (19.3)	
Tumor deposits			< 0.001	< 0.001		< 0.001	0.21		< 0.001
Absent	12,369 (95.2)	4161 (84.1)			421 (67.5)			197 (71.6)	
Present	622 (4.8)	786 (15.9)			203 (32.5)			78 (28.4)	

*Abbreviation*: *CEA* carcinoembryonic antigen, *CRM* circumferential resection margin

^a^ *P*1, comparisons using “classical adenocarcinoma” as the reference. *P*2, comparisons using “adenocarcinoma with mixed subtypes” as the reference

We further compared the clinicopathologic differences between AM and the other two relatively more aggressive histologic subtypes: MAC and SRCC. Compared with MAC, AM was found more frequently in males (*P* = 0.05). AM was also associated with aggressive tumor characteristics including poor differentiation (*P* <  0.001), more advanced T and N stage (*P* <  0.001), distant metastasis (*P* = 0.002), higher positive rates of CRM (*P* <  0.001), perineural invasion (*P* <  0.001), and frequent presence of tumor deposits (*P* <  0.001). As for the comparison between AM and SRCC, AM was associated with better differentiation (*P* <  0.001), distant metastasis (*P* <  0.001), and perineural invasion (*P* = 0.003). No differences were found in other clinicopathologic characteristics between AM and SRCC. Detailed information was shown in Table 2 (*P*2, using “MA” as the reference).

### The prognostic value of histologic subtypes for CRC specific OS

**Fig. 1:**
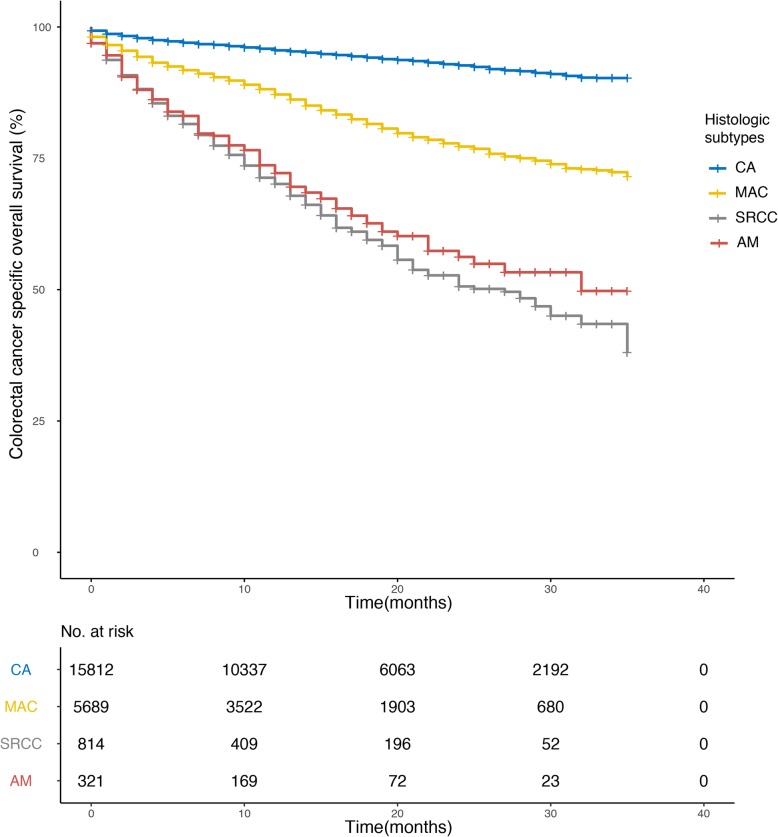
Comparisons of prognosis in histological subtypes plotted with the Kaplan-Meier method

We compared the 3-year CRC specific OS rates between histologic subtypes (Table 3) in the general population and subgroups stratified by TNM stage (0 + I/II/III/IV), tumor location (Colon / Rectum), sex (Male / Female) and age(≤ 66 / > 66). The 3-year OS rates was 90.3 ± 0.004%, 71.6 ± 0.01%, 38.0 ± 0.06% and 49.8 ± 0.06% for CA, MAC, SRCC, and AM, respectively. MAC, SRCC, and AM showed significantly poor survival rates compared with CA. And this difference sustained in most of the subgroups, except for certain TNM stage subgroups. For instance, in stage IV patients, MAC did not show a significant difference in the 3-year OS rate compared with CA. Additionally, there was no obvious difference in the 3-year OS rate when comparing SRCC and CA in stage 0 + I patients. So was when comparing AM and CA in stage II patients. Compared with AM, MAC showed significantly better 3-year OS in the general population as well as in most subgroups, while no prognostic differences were found between AM and SRCC (Table 3, *P*1 using “CA” as the reference, *P*2 using “MA” as the reference). The CRC-specific OS of the four subtypes estimated using the Kaplan-Meier method were shown in Fig. 1. When stratified by TNM stage, AM remained presenting significantly worse CRC-specific OS compared with CA in stage 0 + I, stage III, and stage IV groups (Additional file 2: Figure S1, *P* = 0.04, *P* <  0.001, *P* = 0,001), but not in stage II (Additional file 2: Figure S1, *P* = 0.43).

**Table 3:** Comparisons of the 3-year colorectal cancer specific overall survival rates between classical adenocarcinoma and other histologic types

Characteristics	Classical adenocarcinoma (mean ± SD)	Mucinous adenocarcinoma (mean ± SD)	*P*_1_^a^	*P*_2_^a^	Signet-ring cell carcinoma (mean ± SD)	*P*_1_^a^	*P*_2_^a^	Adenocarcinoma with mixed subtypes (mean ± SD)	*P*_1_^a^
All	90.3 ± 0.4%	71.6 ± 1.3%	< 0.001	< 0.001	38.0 ± 5.8%	< 0.001	0.29	49.8 ± 5.2%	< 0.001
TNM stage									
0 + I	96.3 ± 0.3%	91.0 ± 2.4%	< 0.001	0.32	94.3 ± 4.0%	0.21	0.53	85.9 ± 9.5%	0.03
II	90.2 ± 1.1%	85.5 ± 2.3%	0.04	0.83	71.6 ± 6.1%	< 0.001	0.15	88.7 ± 5.5%	0.43
III	84.1 ± 1.5%	72.0 ± 1.7%	< 0.001	< 0.001	41.4 ± 12.3%	< 0.001	0.51	41.7 ± 10.5%	< 0.001
IV	35.2 ± 2.9%	36.1 ± 2.5%	0.99	< 0.001	9.4 ± 4.8%	< 0.001	0.78	19.5 ± 7.2%	0.001
Tumor location									
Colon	90.2 ± 0.5%	71.8 ± 1.4%	< 0.001	< 0.001	38.4 ± 6.6%	< 0.001	0.54	48.3 ± 5.8%	< 0.001
Rectum	90.4 ± 0.8%	70.4 ± 3.1%	< 0.001	0.10	26.6 ± 12.5%	< 0.001	0.19	56.4 ± 11.9%	< 0.001
Sex									
Male	90.7 ± 0.5%	70.9 ± 2.2%	< 0.001	< 0.001	26.7 ± 11.5%	< 0.001	0.38	51.4 ± 5.2%	< 0.001
Female	89.8 ± 0.6%	72.3 ± 1.3%	< 0.001	< 0.001	45.9 ± 3.8%	< 0.001	0.50	48.8 ± 8.8%	< 0.001
Age (yrs)									
≤ 66	92.9 ± 0.5%	74.7 ± 1.5%	< 0.001	< 0.001	39.8 ± 4.5%	< 0.001	0.12	53.4 ± 6.3%	< 0.001
> 66	86.9 ± 0.7%	68.6 ± 2.0%	< 0.001	< 0.001	39.1 ± 9.4%	< 0.001	0.99	47.8 ± 7.0%	< 0.001

*Abbreviation*: *TNM* tumor-node-metastasis

^a^ *P*1, comparisons using “classical adenocarcinoma” as the reference. *P*2, comparisons using “adenocarcinoma with mixed subtypes” as the reference

We then conducted univariate and multivariate analysis to test the prognostic differences in CRC specific OS between histologic subtypes. By univariate analysis, besides histologic subtypes (*P* <  0.001), other significant prognostic factors including age (≤ 66 / > 66, *P* <  0.001), tumor location (Colon / Rectum, *P* <  0.001), grade (Well differentiated / Moderately differentiated / Poorly differentiated or undifferentiated, *P* <  0.001), TNM stage (0 + I/II/III/IV, *P* <  0.001), race (American Indian/Alaska Native / Asian or Pacific Islander / Black / White, *P* <  0.001), insurance status (Insured / Others, *P* <  0.001), marital status (Married / Widowed / Others, *P* <  0.001), CEA level (Normal / Borderline / Elevated, *P* <  0.001), CRM (Negative / Positive, *P* <  0.001), perineural invasion (Negative / Positive, *P* <  0.001) and tumor deposits (Absent / Present, *P* <  0.001) were identified. All the significant prognostic factors identified by univariate analysis were included for multivariate Cox regression analysis. Factors remained as independent prognostic factors included age (*P* <  0.001), grade (*P* = 0.001), TNM stage (*P* <  0.001), marital status (0.003), CEA (*P* <  0.001), CRM (*P* <  0.001), tumor deposits (*P* <  0.001), and histologic subtype (*P* <  0.001). After adjusting for confounding factors, MAC didn’t have a significantly different prognosis (*P* = 0.20, hazard ratio (*HR*) and 95% confidence interval (95% *CI*): 1.14 (0.93–1.39)), while the inferior prognosis of SRCC and AM remained significant (*P* <  0.001 and *P* = 0.003, *HR* and 95% *CI*: 1.88 (1.37–2.58) and 1.89 (1.25–2.85), respectively) compared with CA (Table 4). In addition, compared with AM, MAC had a significantly better prognosis (*P* = 0.01, *HR* and 95% *CI*: 0.60 (0.40–0.90)), while no survival difference was found between AM and SRCC (*P* = 0.98, *HR* and 95% *CI*: 0.99 (0.65–1.53)) (Table 4).

**Table 4:** Univariate and multivariate analysis for the colorectal-specific overall survival of histologic subtypes in colorectal cancer

Characteristics	Univariate analysis	Multivariate analysis		
	*P* value	*HR*	95% *CI*	*P* value
Sex	0.06			
Male				
Female				
Age (yrs)	< 0.001	2.26	1.87–2.73	< 0.001
≤ 66				
> 66				
Tumor location	< 0.001			
Colon				
Rectum				
Grade	< 0.001			0.001
Well differentiated		1	Reference	
Moderately differentiated		1.50	0.96–2.34	0.08
Poorly differentiated or undifferentiated		2.06	1.30–3.28	0.002
TNM stage	< 0.001			< 0.001
0 + I		1	Reference	
II		2.04	1.24–3.35	0.01
III		5.12	3.23–8.09	< 0.001
IV		15.39	9.55–24.79	< 0.001
Race	< 0.001			
American Indian/Alaska Native				
Asian or Pacific Islander				
Black				
White				
Insurance status	< 0.001			
Insured				
Others				
Marital status	< 0.001			0.003
Married		1	Reference	
Widowed		1.40	1.10–1.78	0.01
Others		1.33	1.08–1.63	0.01
CEA	< 0.001			< 0.001
Normal		1	Reference	
Borderline		1.43	0.45–4.51	0.54
Elevated		1.76	1.44–2.15	< 0.001
CRM	< 0.001	1.47	1.22–1.77	< 0.001
Negative				
Positive				
Perineural invasion	< 0.001			
Negative				
Positive				
Tumor deposits	< 0.001	1.70	1.39–2.08	< 0.001
Absent				
Present				
Histologic subtype (classical adenocarcinoma as reference)	< 0.001			< 0.001
Classical adenocarcinoma		1	Reference	
Mucinous adenocarcinoma		1.14	0.93–1.39	0.20
Signet-ring cell carcinoma		1.88	1.37–2.58	< 0.001
Adenocarcinoma with mixed subtypes		1.89	1.25–2.85	0.003
Histologic subtype (adenocarcinoma with mixed subtypes as reference)	< 0.001			< 0.001
Adenocarcinoma with mixed subtypes		1	Reference	
Classical adenocarcinoma		0.53	0.35–0.81	0.003
Mucinous adenocarcinoma		0.60	0.40–0.90	0.01
Signet-ring cell carcinoma		0.99	0.65–1.53	0.98

*Abbreviation*: *TNM* tumor-node-metastasis, *CEA* carcinoembryonic antigen, *CRM* circumferential resection margin

## Discussion

In this study, we compared the clinicopathologic and survival differences of CA with two previously widely investigated histologic subtypes, MAC and SRCC. Both subtypes were significantly associated with more aggressive features compared with CA, while only SRCC showed significantly poorer survival. Furthermore, we also focused on another rare histologic subtype, AM, whose frequency is about 2/5 of SRCC. We identified AM as a subgroup significantly associated with more advanced tumor grade and stage, as well as worse survival compared with CA. In addition, the aggressiveness of AM was between MAC and SRCC according to the clinicopathologic associations. The prognosis of AM was found comparable with SRCC and worse than MAC.

Our conclusions about the clinicopathologic relevance of MAC and SRCC were mainly in consistency with previous reports. They were associated with poorer tumor grade [19, 28–30], deeper primary tumor invasion [19, 22, 28], regional lymph nodes metastasis [19, 28], more advanced TNM stage [29, 30], and higher level of CEA [22]. Both MAC and SRCC have been repeatedly reported to be less common in the rectum [19, 22, 29, 30]. MAC was found more frequently in females [30]. We also demonstrated the relevance of MAC and SRCC with several postoperative features, including CRM, perineural invasion and tumor deposits. These factors had been found to be associated with poor survival [16, 17, 31]. From the above, the prognostic value of histologic subtypes might be confounded by these prognostic factors. Interestingly, although MAC was significantly associated with aggressive tumor features and advanced tumor stage, its survival difference from CA was not independently significant according to multivariate analysis. The prognostic value of MAC has been controversial. Several studies reported MAC to be an independent negative prognostic factor [32, 33]. However, most of the other reports were in accordance with our conclusions [19, 22, 23]. MSI-H, more frequently found in MAC, was identified as a positive prognostic marker in CRC [24, 25]. This might partially explain the discordance between the clinicopathologic and prognostic relevance of MAC.

The consistency of clinicopathologic and prognostic relevance of SRCC highlighted a potential distinct aggressive tumor biology mechanism. Previous studies speculated that SRCC might arise from different cell origins compared with CA [34]. This distinct aggressive histologic subtype might benefit from intensified systemic therapy [26] and closer follow-up. Most importantly, our study identified that AM had a poor prognosis relative to SRCC. This subtype has not been well documented in the literature of CRC. In lung cancer, AM exhibited a greater genetic heterogeneity of EGFR mutation and ALK rearrangement. Thus, both intrinsic vicious biology and high heterogeneity might contribute to the aggressiveness and refractory of AM. Immunohistochemistry might be helpful for identification of tumor components [35], which should be considered when selecting systemic chemotherapy regimens.

The main limitation of our study is that this is a retrospective analysis of patients using the SEER database. The majority of patients in the SEER database were defined as adenocarcinoma NOS and were not included in the analysis. This could possibly cause some biases. Thus the results need to be validated in a more precise database.

## Conclusions

Our study not only confirmed the clinicopathologic and survival differences of CA with MAC and SRCC with a large-sized sample, but also identified another histologic subgroup with aggressive tumor features and poor prognosis. The relatively large study population and the data source of the SEER database made the conclusions quite credible. However, there was no available information on DFS and genetic alterations, lack of such information is a limitation of our study.

## Supplementary information

**Additional file 1 **: **Table S1.** The frequency distribution of ICD-O-3 codes and histologic types in colorectal adenocarcinoma.

**Additional file 2: Figure S1.** Comparisons of prognosis in histological subtypes stratified by TNM stage.

## Data Availability

The data were retrieved from publicly accessible database “Surveillance, Epidemiology, and End Results” (SEER), the website is “https://seer.cancer.gov/”. The definite data used in this study is available from the corresponding author on reasonable request.

## Abbreviations

AJCC: American Joint Committee on Cancer
ALK: Anaplastic lymphoma kinase
AM: Adenocarcinoma with mixed subtypes
CA: Classic adenocarcinoma
CEA: Carcinoembryonic antigen
CRC: Colorectal cancer
CRM: Circumferential resection margin
EGFR: Epidermal growth factor receptor
ICD-O-3: 3rd edition of the International Classification of Diseases for Oncology
MAC: Mucinous adenocarcinoma
MSI-H: High microsatellite instability
NCCN: National Comprehensive Cancer Network
SEER: Surveillance, Epidemiology and End Results
SRCC: Signet-ring cell carcinoma
TNM: Tumor-node-metastasis
