# Supplementary material for: Adenocarcinoma with mixed subtypes is a rare but aggressive histologic subtype in colorectal cancer
Source: BMC Cancer. 2019 Nov 8;19:1071. doi: 10.1186/s12885-019-6245-5 (PMC6842229; doi:10.1186/s12885-019-6245-5)
Supplement: Supplementary file 1 — Additional file 1 : Table S1. The frequency distribution of ICD-O-3 codes and histologic types in colorectal adenocarcinoma. [file 12885_2019_6245_MOESM1_ESM.docx]

Additional file 1: Table S1. The frequency distribution of ICD-O-3 codes and histologic types in colorectal adenocarcinoma.

| **Codes** | **Histologic type** | **Number (%)** | **Frequency distribution** |
| --- | --- | --- | --- |
| 8140 | Adenocarcinoma, NOS | 49,131 (68.4) | - |
| 8141 | Scirrhous adenocarcinoma | 2 (< 0.1) | < 0.1 |
| 8143 | Superficial spreading adenocarcinoma | 2 (< 0.1) | < 0.1 |
| 8144 | Adenocarcinoma, intestinal type | 23 (< 0.1) | 0.1 |
| 8145 | Adenocarcinoma, diffuse type | 3 (< 0.1) | < 0.1 |
| 8210 | Adenocarcinoma in adenomatous polyp | 6,493 (9.0) | 28.6 |
| 8211 | Tubular adenocarcinoma | 63 (0.1) | 0.3 |
| 8220 | Adenocarcinoma in adenomatous polyposis coli | 28 (< 0.1) | 0.1 |
| 8221 | Adenocarcinoma in multiple adenomatous polyps | 10 (< 0.1) | < 0.1 |
| 8255 | Adenocarcinoma with mixed subtypes | 321 (0.4) | 1.4 |
| 8260 | Papillary adenocarcinoma, NOS | 15 (< 0.1) | < 0.1 |
| 8261 | Adenocarcinoma in villous adenoma | 1,750 (2.4) | 7.7 |
| 8262 | Villous adenocarcinoma | 44 (0.1) | 0.2 |
| 8263 | Adenocarcinoma in tubulovillous adenoma | 7,379 (10.3) | 32.5 |
| 8480 | Mucinous adenocarcinoma | 5,068 (7.1) | 22.3 |
| 8481 | Mucin-producing adenocarcinoma | 621 (0.9) | 2.7 |
| 8490 | Signet-ring cell carcinoma | 814 (1.1) | 3.6 |
| 8574 | Adenocarcinoma with neuroendocrine differentiation | 43 (0.1) | 0.2 |
