# Supplementary figures and images for: Adenocarcinoma with mixed subtypes is a rare but aggressive histologic subtype in colorectal cancer
Source: BMC Cancer. 2019 Nov 8;19:1071. doi: 10.1186/s12885-019-6245-5 (PMC6842229; doi:10.1186/s12885-019-6245-5)

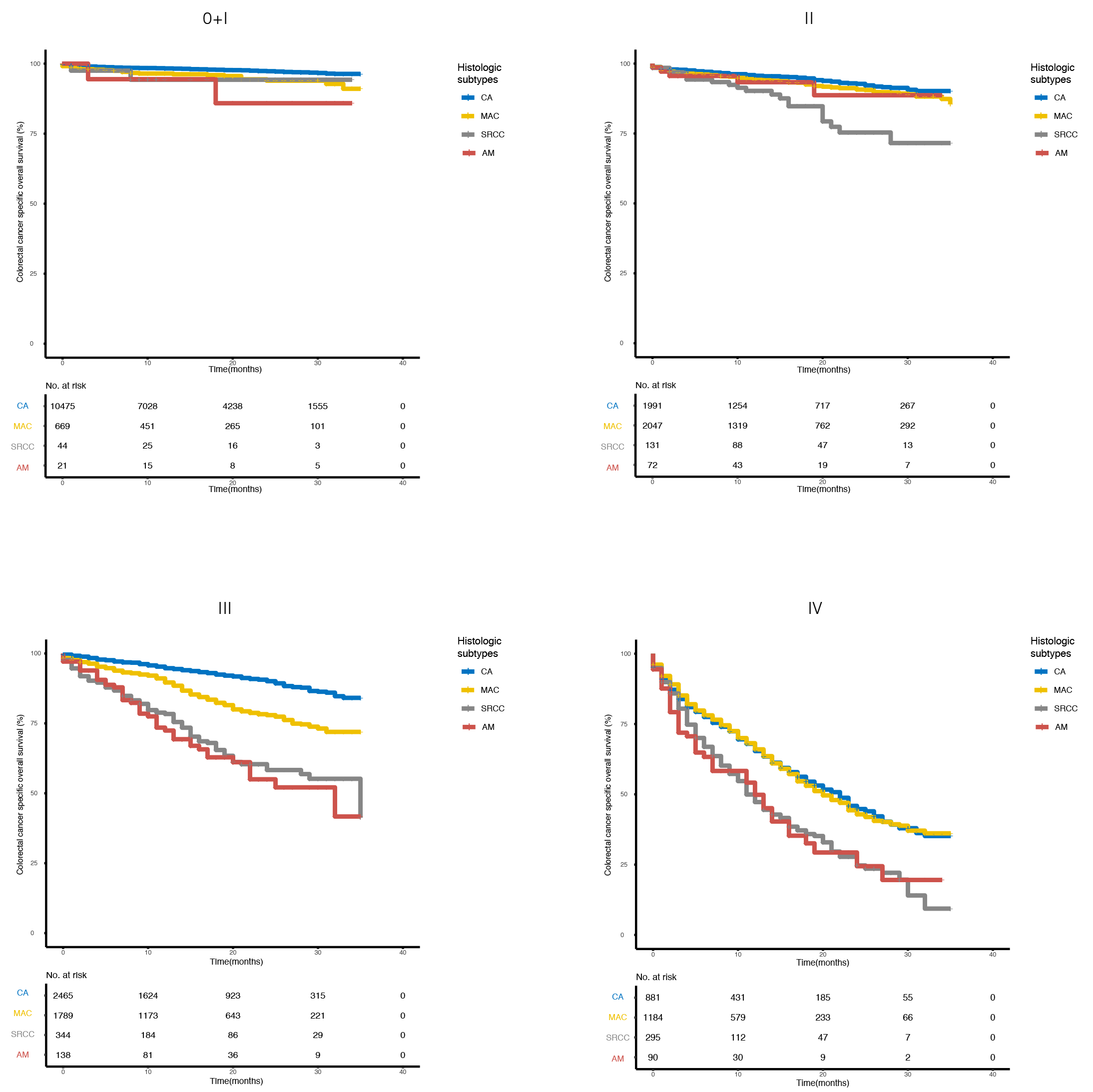

Supplement: Supplementary file 2 — Additional file 2: Figure S1. Comparisons of prognosis in histological subtypes stratified by TNM stage. [file 12885_2019_6245_MOESM2_ESM.tif]
